# Accuracy and Powder Removal Limits in Multi Jet Fusion 3D Printing

**DOI:** 10.3390/polym17202804

**Published:** 2025-10-21

**Authors:** Karel Raz, Zdenek Chval, Petra Faitova

**Affiliations:** Faculty of Mechanical Engineering, Regional Technological Institute, University of West Bohemia, Univerzitni 2732/8, 301 00 Plzen, Czech Republic; zdchval@fst.zcu.cz (Z.C.); hofrichp@fst.zcu.cz (P.F.)

**Keywords:** MJF, nylon, lattice, accuracy, 3D printing

## Abstract

Multi Jet Fusion (MJF) is a leading technology for producing functional polymer parts. However, it still faces challenges with dimensional accuracy and removing unfused powder from complex internal geometries. First, dimensional accuracy was mapped by producing 45 identical PA12 specimens on an HP MJF 4200 printer in a 5 × 9 layout across five vertical layers. The analysis revealed a consistent pattern: parts located in the central positions of the build volume exhibited the poorest accuracy, while those near the perimeter were the most precise, regardless of their vertical height. This spatial variation is attributed to non-uniform thermal control from the printer’s adaptive lamp–thermal camera system. Second, the limits of powder removal from closed body-centered cubic (BCC) lattice structures were quantified. Using sandblasting and X-ray inspection, a strong inverse relationship was found between a lattice’s relative density and the maximum thickness that could be thoroughly cleaned of powder. For example, low-density structures (ρ = 0.07) could be cleaned up to five layers deep, whereas high-density structures (ρ = 0.39–0.47) were limited to only 1.5–1.7 layers. These findings offer actionable guidelines for optimizing part placement and designing internal lattice structures for MJF technology. The key findings are the spatial variation in dimensional accuracy in MJF printing, where the central parts are the least accurate and perimeter parts are the most precise, and the inverse relationship between a lattice’s relative density (ρ) and cleanable thickness. Specifically, low-density structures (ρ = 0.07) could be thoroughly cleaned up to five layers, while high-density ones (ρ = 0.39–0.47) were limited to approximately 1.5–1.7 layers. The layer thickness was a pre-designed parameter (2, 3, 4, and 5 layers), and powder removal was supported by using automated sandblasting followed by verification via industrial X-ray imaging.

## 1. Introduction

Additive manufacturing (AM) has progressed from a prototyping method to a mature production technology capable of delivering functional parts [1,2]. Its key advantages include design freedom [3], efficient material use [4], and the ability to fabricate complex geometries with integrated features [5,6]. Within polymer-based AM, powder bed fusion (PBF) processes—especially Selective Laser Sintering (SLS) and Multi Jet Fusion (MJF)—are among the most widely adopted for engineering polymers such as polyamide 12 (PA12). MJF is unique in that it employs inkjet nozzles to distribute fusing and detailing agents [7], followed by infrared heating. This approach enables high throughput and delivers parts with competitive surface quality [8,9].

Despite these benefits, AM processes still face inherent challenges. Dimensional accuracy is strongly influenced by part orientation, thermal gradients, and build position within the chamber [10,11]. Published studies [12,13] demonstrate that spatial variability frequently introduces systematic deviations in geometry and size, while other works [14,15] highlight the impact of scan strategy, layer thickness, and build orientation on both accuracy and mechanical performance [16]. Furthermore, issues such as surface roughness, anisotropic mechanical behavior, and residual stress remain persistent across AM technologies [17].

For MJF specifically, prior research has concentrated mainly on mechanical properties, powder reuse, and surface finish [18,19]. Considerably fewer studies have investigated location-dependent accuracy within the build volume [20] or the efficiency of powder removal from enclosed lattice structures [21,22,23]. These aspects are critical in industrial practice, where predictable tolerances and complete removal of unfused powder from internal cavities are essential to ensure both performance and reliability.

The present work aims to address these gaps. By linking process constraints with design strategies, this study provides practical guidelines for part placement and lattice configuration to enhance predictability and performance in MJF production [24,25].

## 2. Materials and Methods

The two primary limitations of the MJF process are dimensional accuracy and powder removal efficiency. To evaluate positional accuracy within the build volume, 45 identical specimens were printed on an HP (Palo Alto, CA, USA) MJF 4200 system. Each part was oriented at 20° to the xy-plane and 10° to the yz-plane, arranged in a 5 × 9 grid spanning the entire build plate and distributed over five vertical layers [26]. After printing, all parts were cooled inside the machine using the standard MJF cycle to minimize thermal stress and deformation [27].

Dimensional accuracy was assessed using a calibrated digital caliper and optical measurement system, with deviations determined by comparing measured and nominal CAD dimensions. The results were mapped to specific build coordinates to identify position-dependent accuracy trends.

The second part of the study examined powder-removal limits in closed BCC lattice structures enclosed by solid walls with defined vent openings. The relative density (ρ) was varied by changing the strut diameter, and four structure thicknesses (2–5 layers in the z-direction) were produced for each density. Powder removal was performed using automated sandblasting at a pressure of 4.4 bar and a standoff distance of 400 mm [28,29], with a constant blasting duration applied to all samples. The residual powder was evaluated using industrial X-ray imaging to confirm both complete and partial depowdering in otherwise inaccessible internal regions.

## 3. The Influence of Position and Settings of the 3D Printing Process on Accuracy

This part of the research aims to gain a deeper understanding of and describe the behavior of the material during the printing process using MJF technology. The transferability of the described methodology to other manufacturing technologies is obvious. The aim is to tell the cooling process and subsequent deformation, so that the knowledge gained can be applied in the design of the part, and the reason for any difference between the simulated (predicted) and actual deformation can be explained [30]. Residual heat is generated in the printing process [31], and heat is also used to sinter the material [32]. This results in deformation that can negatively affect the dimensional stability of the printed part [33,34]. By leveraging knowledge of the production and cooling processes, it is possible to accurately position parts that require higher precision, thereby minimizing deformation of these parts [35,36].

**Figure 1:**
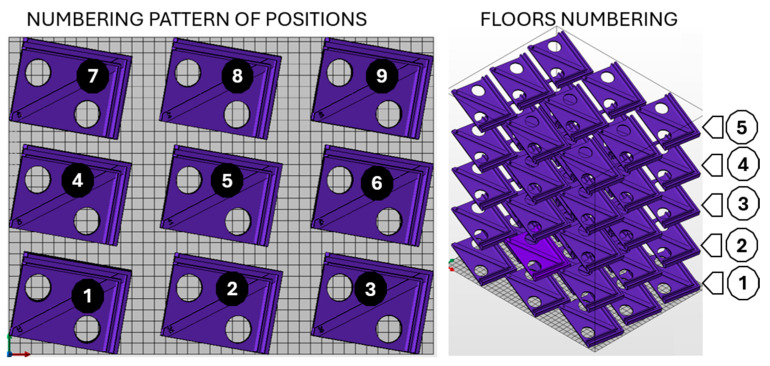
Numbering of specimens in the printing area, (**left**)—floor 1, (**right**)—overall view.

As part of the analysis, 45 samples were distributed in the print space of the HP MJF 4200 printer, measuring 380 × 284 × 380 mm. These samples were arranged in 5 layers of 9 and oriented in the printing space at an angle of 20° to the xy plane and 10° to the yz plane, as shown in Figure 1. The number of specimens was chosen based on their size and the ease of measuring deformations.

Samples were numbered schematically as shown in Figure 1. The first floor is numbered 1–9, with the first sample being closest to the printer’s coordinate system and the last one being furthest away. The second floor has samples numbered 10–18, using the same system as the first floor, with sample number 10 being closest to the printer’s coordinate system and sample 18 being furthest away. The other floors are numbered similarly.

**Figure 2:**
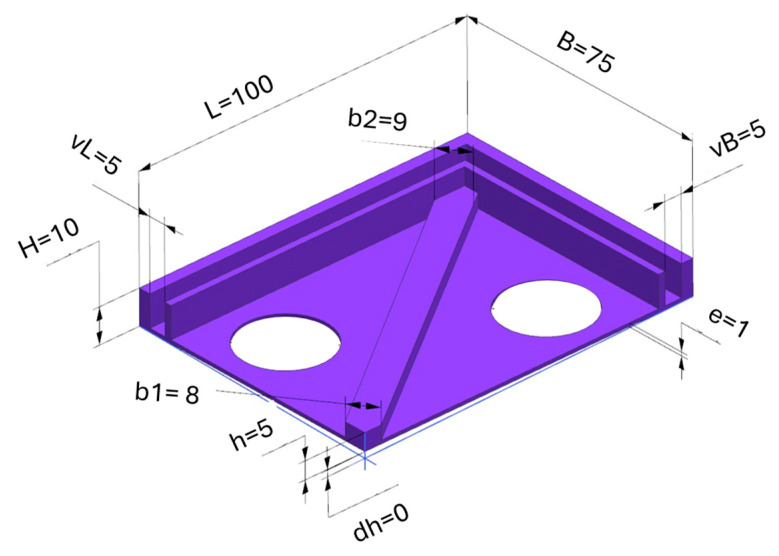
Measured and analyzed dimensions selected for analysis.

After printing the samples, the dimensions shown in Figure 2 were measured. The part was designed as a plate cover with a one-sided rib. The stacking angle and position were according to the official HP software (Version 2.0) procedure. Due to printing inaccuracies and shrinkage of the sample during cooling, there was a slight twisting of the part, which is marked as δh and should ideally be zero in an ideal state. There was also an overall shrinkage of the part, which is most noticeable in the changes in its width and length.

**Figure 3:**
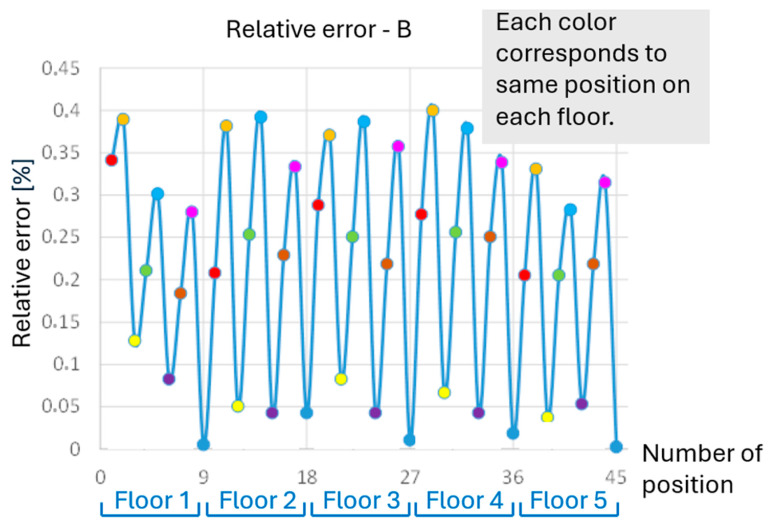
Relative measured error for dimension B—example for one dimension.

The analysis of one selected dimension is presented below in Figure 3, specifically the width of part B. The overall analysis of accuracy based on the deviations of all dimensions is shown later.

**Figure 4:**
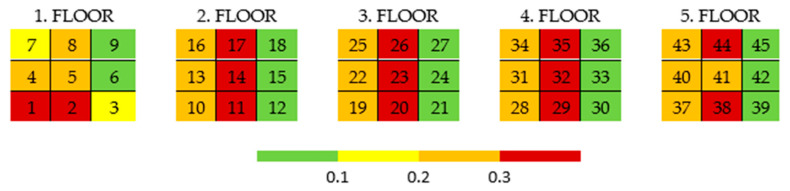
Accuracy scale for each position with respect to the B dimension (example).

The measured dimension B does not show significant differences in the accuracy of elements in individual floors. However, the greatest accuracy is always achieved by elements located in the corner positions. The worst accuracy is achieved on each floor in the middle row. Everything is evident from Figure 3 and Figure 4. The exact position of parts at all heights (on all floors) is always represented by the same color, which has a recurring effect on accuracy.

The accuracy of the B dimension for individual positions (quadrants) in the printer’s print space can be schematically illustrated in Figure 4, where each table represents one floor, and the color indicates the accuracy of the given position. Again, the repetition of the overall pattern across the individual floors is obvious. The total deviation from the required dimension was divided into four color-coded levels.

**Figure 5:**
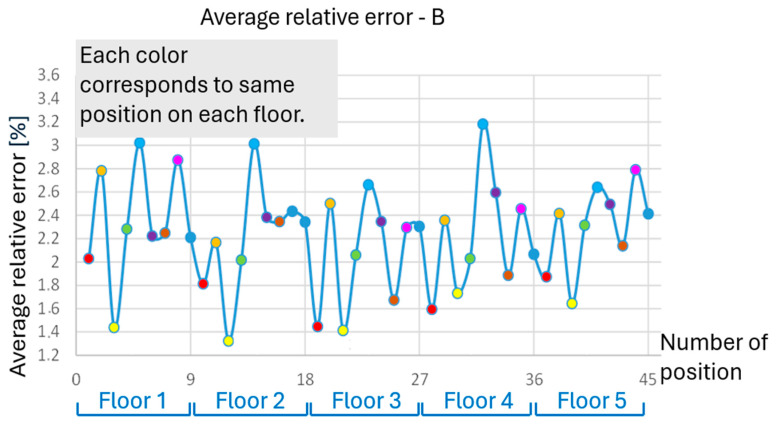
Average relative error with respect to the position (floor)—overall value.

To understand the overall behavior of the parts and describe the deformations in all directions, a summary analysis of all measured dimensions is conducted. After processing the measurements of all dimensions, it is possible to determine the average relative error of the samples depending on their position. Figure 5 illustrates the average relative error of all measured dimensions for each position. The worst accuracies are demonstrated by the middle positions, 5, 14, 23, 32, and 41, as well as positions 8, 2, and 44. These positions should be avoided when printing parts requiring high accuracy. On the other hand, the best average results are achieved by the extreme positions, specifically at positions 12, 21, 3, and 19, and followed by positions 28, 39, 25, and 30.

**Figure 6:**
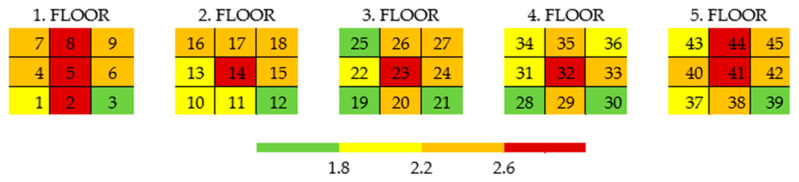
Accuracy of specimens with respect to the position in the printing area.

The overall accuracy of individual positions (quadrants) in the printer’s print space can be schematically illustrated in Figure 6, where each table represents one floor and the color indicates the accuracy of the corresponding position.

Generally, the deviation across the build plate was described and considered to be 0.18 mm, with a standard deviation of 0.07 mm. Central regions showed a deviation of up to 0.25 mm, while edge regions averaged 0.12 mm. Measurements were repeated three times per dimension using a digital caliper (±0.01 mm) and an optical setup with a precision of ±0.02 mm; the overall uncertainty was ±0.03 mm.

Dimensional deviations were found to be mainly contractile, indicating uniform shrinkage during cooling. The most significant errors occurred along the x-axis, moderate errors along the y-axis, and the least significant errors along the z-axis, indicating a slight directional dependence. Central positions exhibited approximately 30–40% higher shrinkage than peripheral ones. This confirms that positional effects are systematic rather than random across the build plate.

In conclusion, the behavior observed during the printing process can be inferred from the previous results. The movement of the print heads in the longitudinal direction of the printer has a negligible effect on overall accuracy. The state of the print heads does not play a role either. In the analysis, printing lamps with a difference in operating time of the print heads of up to 200% were used, which did not show differences in the parts connected to the given head.

**Figure 7:**
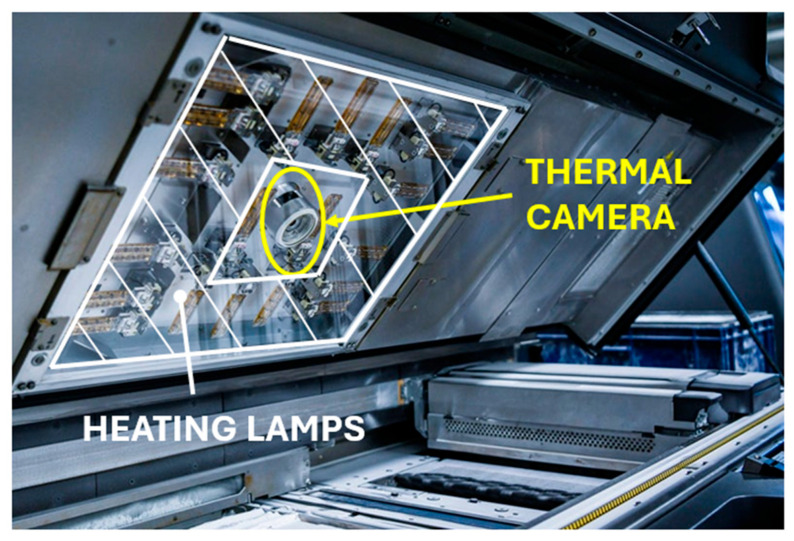
MJF 4200 printer—location of heating lamps and thermal camera in the AM machine cover.

The worst accuracy is for parts located in the central area of the printing space, regardless of height. This effect is caused by the non-uniform distribution of temperature from the lamps. These lamps are adaptively controlled based on recordings from the thermal camera, ensuring a uniform temperature. It is clear that in the middle area, where the thermal camera (and not the lamp, see Figure 7) is located, the control of this temperature is worse, and therefore this position has the worst accuracy. This figure is crucial for understanding the source of heat during the printing process. Generally, the estimated temperature difference between the center and edges is approximately 5–10 °C, leading to local shrinkage variations of 0.05–1%. This gradient is sufficient to cause the observed dimensional inaccuracies of 0.1–0.2 mm in central positions.

The machine does not display the temperature report to the user, as it is a closed system. There is only a simple thermal camera view, which is not easy to reproduce. In the future, machines should be equipped with a more precise thermal control system that is open to user input.

## 4. Limits of Lattice Structures in MJF Printing—Problems with Internal Powder Removal

One of the advantages of MJF technology is the ability to print without support structures [37]. With this technology, it is possible to use arbitrarily oriented internal fillings (it is not necessary to refer to the rules for printing without supports, as is the case with FDM technologies and the use of standard lattice structures) to relieve the parts [38,39].

However, with a closed volume, there is a problem with removing non-solid powder and achieving the primary goal of printing with internal cavities, which is to make the part lighter [40]. It is therefore necessary to determine the geometric rules for penetrations through the outer envelope surface of parts and the design of internal reinforcement structures [41] so that the removal of excess powder is possible and the requirement for weight reduction is met [42].

**Figure 8:**
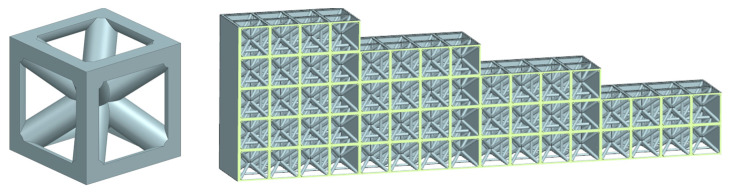
Tested sample from BCC lattice cell—CAD model, (**left**)—one lattice cell, (**right**)—cross-section.

In this part of the work, a set of test samples was created to determine the optimal approach to designing the filling of internal cavities and penetrations in the envelope surface of the part for removing excess powder. A BCC type lattice was chosen for the internal structure, as shown in Figure 8, which can be oriented arbitrarily.

For the research, samples with different relative densities of the lattice structure were printed, and each sample was graded for thicknesses ranging from 2 to 5 layers.

The BCC lattice was selected because it is the most commonly used topology in MJF for lightweight structures, providing isotropic load paths. Other topologies, such as FCC or hexagonal lattices, generally feature smaller interconnect angles and narrower flow channels, which would reduce powder removal efficiency under identical venting and blasting conditions. For reproducibility, the BCC unit cell used in this study had an edge length of 5 mm, and the reported relative densities were calculated from this constant cell size and variable strut diameters.

The relative density depends on the diameter of the rod (which changes linearly) within the BCC lattice structure, and it is the ratio between the volume of an individual cell and the volume of a whole cube. The list of relative densities of the lattice structures of the printed samples is shown in Table 1. All parameters were selected based on the experimental setup for the given conditions.

**Figure 9:**
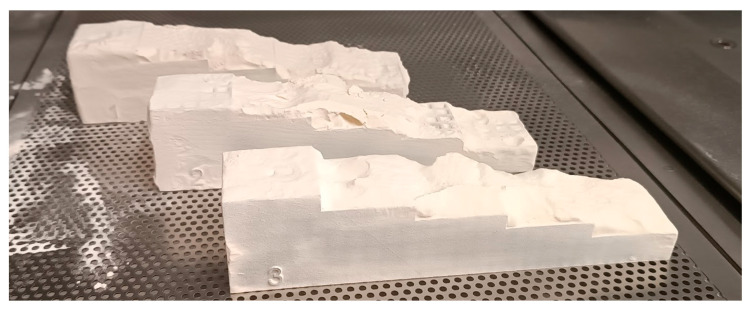
Specimens before sandblasting, after the AM production process.

After printing the samples (see Figure 9), they were sandblasted with a pressure of approximately 4.4 bar using a nozzle from a distance of 400 mm.

**Figure 10:**
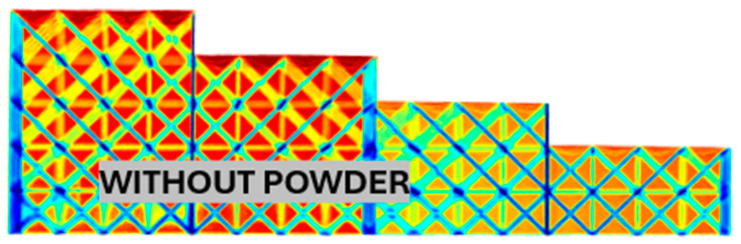
Thickness of the sandblasted layer for relative cell density 0.07, rod thickness 1 mm.

After sandblasting, the samples were evaluated. While, for example, for a rod thickness of 1 mm and a relative density of 0.07 (see the following Figure 10), a complete evaluation could be performed visually (the powder was removed entirely), the other samples were evaluated based on their X-ray images.

The sandblasting parameters (4.4 bar, 400 mm distance) were selected based on preliminary tests that confirmed complete powder removal without strut damage, aligning with industrial depowdering guidelines for PA12 MJF parts. This setting ensured consistent comparison across samples. Alternative methods, such as ultrasonic cleaning or vacuum-assisted air blasting, could enhance powder removal in dense lattices with narrow pores; however, they may risk incomplete drying or surface erosion. These approaches could complement sandblasting for complex or high-density BCC structures.

Figure 10 shows an X-ray image of a rod with a thickness of 1 mm, corresponding to a relative density of 0.07. This image serves as a reasonable verification of the visual method, as it is clearly visible that all thicknesses were sandblasted perfectly. For the relative density of the rod, it is therefore possible to sandblast at least five layers.

**Figure 11:**
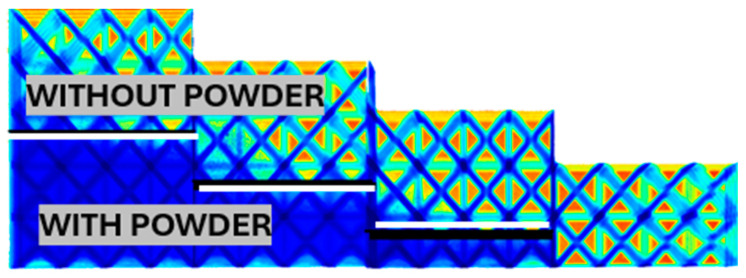
Thickness of the sandblasted layer for relative cell density 0.18, rod thickness 1.66 mm.

For other samples, the blast depth was no longer apparent without X-rays. For example, for a sample with a rod diameter of 1.66 mm and a relative density of 0.18, it was possible to blast the first two layers completely. In contrast, for other thicknesses of 3, 4, and 5 layers, the layers were only partially blasted to a depth of approximately 2.6 layers. The blasted limit is marked with a thick white line (Figure 11).

**Figure 12:**
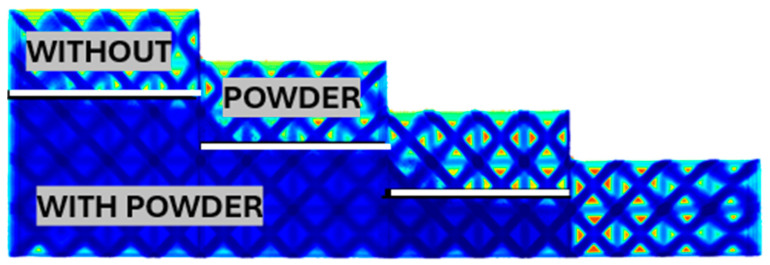
Sandblasted layer thickness for relative cell density 0.39, rod thickness 2.66 mm.

With further increases in relative density, the thickness of the sandblasted layer decreases, as shown in Figure 12. For a rod diameter of 2.66 and a relative density of 0.39, it is possible to completely sandblast a part of the sample with a thickness of 2 layers. However, for more layers, it was not possible to achieve sandblasting of more than 1.6 layers. A thick white line again highlights the interface of the sandblasted part.

The results indicate a decreasing dependence of the relative density on the sandblasted thickness, as shown in Table 2.

**Figure 13:**
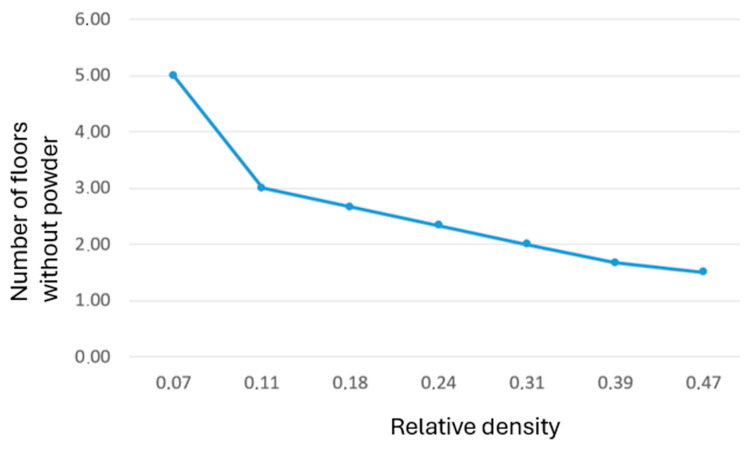
Dependence of the thickness of sandblasted layers on the relative cell density.

This result can be illustrated graphically (Figure 13), which shows a decrease in the dependency of the sandblasted layers on the relative density of the BCC lattice cell.

## 5. Discussion

The experimental mapping of dimensional accuracy across the MJF build volume confirmed that spatial variability is a consistent and reproducible phenomenon [43]. The poorest accuracy was repeatedly observed in the central positions of each floor (e.g., positions 5, 14, 23, 32, and 41), while edge positions generally had the most minor deviations. This finding aligns with previous reports on 3D printing systems, where non-uniform thermal fields and cooling rates have been shown to affect dimensional fidelity [44,45]. In our case, the cause is attributed to the adaptive heating system of the HP MJF 4200, which regulates lamp output via a centrally located thermal camera. The central region’s more challenging thermal control likely results in localized overheating or underheating, leading to uneven shrinkage during cooling.

Notably, the observed inaccuracy patterns were largely independent of part height, indicating that horizontal location plays a more critical role than vertical build position. This suggests that designers and production engineers can consider tolerance issues by preferentially placing essential parts of peripheral zones of the build chamber. The AM machine should be equipped with a thermal output in the future to validate the thermal resolution.

The problem with removing the internal powder from parts showed a relationship between relative density and maximum cleanable thickness. At low densities (ρ ≈ 0.07), complete powder removal was achievable through up to five BCC layers, while at higher densities (ρ ≈ 0.39–0.47), only 1.5–1.7 layers could be fully cleaned. These results are consistent with findings in lattice manufacturing studies [46,47]. The industrial implication is that, for closed MJF lattices, vent design must be coupled with density and layer-thickness limitations to avoid trapped powder compromising mass reduction goals. The research has to be also focused on different types of lattice structures in the future. The BCC was chosen, because it is the most often used type of the lattice cell.

Our findings on central accuracy loss agree with [13,40], who reported similar spatial deviations in MJF and SLS builds due to uneven thermal gradients. The powder-removal behavior follows trends observed by [21,40], where higher lattice density reduced depowdering depth. Compared with these studies, our work extends the analysis by linking positional accuracy and lattice density to specific design recommendations for MJF production.

## 6. Conclusions

This study investigated two critical but underexplored limitations of Multi Jet Fusion technology: spatial variation in dimensional accuracy and powder removal constraints in closed lattice structures. The key conclusions are following. Dimensional accuracy varies systematically across the MJF build plate. Peripheral positions yield the most accurate dimensions, while central locations show the largest deviations, independent of height. This effect is likely linked to non-uniform heating control in the printer lid.

Powder removal in closed BCC lattices is strongly dependent on relative density and thickness. Low-density structures (ρ ≈ 0.07) allow full cleaning up to five layers, while high-density lattices (ρ ≈ 0.47) are limited to approximately 1.5 layers.

Design and production guidelines emerge directly from these findings. Critical tolerance parts should be located near chamber edges, and lattice designs should limit closed thickness according to density to ensure complete depowdering.

By quantifying these process–geometry interactions, this work provides actionable guidance for part placement and design-for-MJF strategies. The methodology can be extended to other powder-bed fusion systems to map accuracy variation and establish practical limits for internal structure cleaning.

## Figures and Tables

**Table 1:** Relative density with respect to the rod diameter.

Rod Diameter of the Rod [mm]	Relative Density [-]
1	0.07
1.33	0.11
1.66	0.18
2	0.24
2.33	0.31
2.66	0.39
3	0.47

**Table 2:** Thickness values of sandblasted layers at different relative values.

Relative Density	Thickness of Sandblasted Layers
0.07	5.00
0.11	3.00
0.18	2.67
0.24	2.33
0.31	2.00
0.39	1.67
0.47	1.50

## Data Availability

Not applicable.

## Abbreviations

The following abbreviations are used in this manuscript:

MJF	Multi Jet Fusion
HP	Hewlett Packard
AM	Additive Manufacturing
PA	Polyamide
